# Comparative transfection of DNA into primary and transformed mammalian cells from different lineages

**DOI:** 10.1186/1472-6750-10-9

**Published:** 2010-02-08

**Authors:** Rosalie Maurisse, David De Semir, Hamid Emamekhoo, Babak Bedayat, Alireza Abdolmohammadi, Hooman Parsi, Dieter C Gruenert

**Affiliations:** 1 California Pacific Medical Center Research Institute, San Francisco, CA, USA; 2 Department of Laboratory Medicine, University of California San Francisco, San Francisco, CA, USA; 3 Department of Medicine, University of Vermont, Burlington, VT, USA; 4 Current address: Medicen, 6 rue Alexandre Cabanel, 75015 Paris, France; 5 Current address: Department of Anesthesiology and Critical Care, Massachusetts General Hospital, Harvard Medical School, Boston, MA, USA; 6 Current address: Department of Internal Medicine, Good Samaritan Hospital, Cincinnati, OH, USA

## Abstract

**Background:**

The delivery of DNA into human cells has been the basis of advances in the understanding of gene function and the development of genetic therapies. Numerous chemical and physical approaches have been used to deliver the DNA, but their efficacy has been variable and is highly dependent on the cell type to be transfected.

**Results:**

Studies were undertaken to evaluate and compare the transfection efficacy of several chemical reagents to that of the electroporation/nucleofection system using both adherent cells (primary and transformed airway epithelial cells and primary fibroblasts as well as embryonic stem cells) and cells in suspension (primary hematopoietic stem/progenitor cells and lymphoblasts). With the exception of HEK 293 cell transfection, nucleofection proved to be less toxic and more efficient at effectively delivering DNA into the cells as determined by cell proliferation and GFP expression, respectively. Lipofectamine and nucleofection of HEK 293 were essentially equivalent in terms of toxicity and efficiency. Transient transfection efficiency in all the cell systems ranged from 40%-90%, with minimal toxicity and no apparent species specificity. Differences in efficiency and toxicity were cell type/system specific.

**Conclusions:**

In general, the Amaxa electroporation/nucleofection system appears superior to other chemical systems. However, there are cell-type and species specific differences that need to be evaluated empirically to optimize the conditions for transfection efficiency and cell survival.

## Background

Numerous chemical and physical methods have been used to introduce DNA expression vectors into mammalian cells both *in vitro *and *in vivo*, including, but not limited to, calcium phosphate precipitation, microinjection, electroporation, receptor-mediated gene transfer, particle guns, viral vectors, polyfection and lipofection [1].

The use of cationic liposome/DNA complexes (lipoplexes) and cationic polymers/DNA (polyplexes) for the transfer of genes into somatic cells has become very popular due to its limited toxicity and relative effectiveness *in vitro*. The ionic interaction between cationic lipids and DNA leads to the formation of lipoplexes that are generally slightly cationic. The resulting DNA/lipid complexes fuse with the anionic cytoplasmic membrane and/or are introduced into the cells via an endocytic pathway [2]. The delivery of the DNA into the nucleus is still not fully understood. While transfection with cationic lipids and polymers offers some advantages over viral transduction, such as simplicity of production, low toxicity, and low immunogenicity; it has yet to reach the levels observed with viral transduction. Furthermore, the adherence of the cationic complexes to the nucleic acid can interfere with its accessibility to enzymes required for processing the DNA [3].

One of the most effective and accessible physical transfection methods, electroporation (also known as electrotransfer, electropermeabilization, or nucleofection), involves the application of brief electric pulses to cells or tissues to increase the permeability of cells to macromolecules [1,4]. The recent development of the nucleofection system has been a significant advance over standard electroporation systems that have been limited by high toxicity and a requirement for large numbers of cells. A number of cell lines have already been tested for their compatibility with the nucleofection system [5-12]. However, there have been no systematic studies comparing nucleofection to chemical transfection systems in various cell types across species.

In this study, chemical reagent-mediated transfection was compared to nucleofection using a number of primary and immortalized cell systems in three different mammalian species (human, rabbit, and pig) to evaluate the efficiency and toxicity. The results presented here indicate that nucleofection is more effective than chemical transfection reagents from several different cationic categories (dendrimer, polyethylenimine, lipid) at delivering DNA into a variety of different cell types. These studies also provided useful insight into transfection optimization conditions and relative cell viability for the various cells tested.

Previous studies indicated that the ratio of DNA to lipid is an important variable that determines the efficiency of transfection and the cellular toxicity [1,13]. To evaluate the effect of varying the ratio of DNA to transfection reagent, the cells were transfected with a constant quantity of plasmid DNA in a complex with a variable amount of a given transfection reagent. One to three different DNA/reagent ratios were evaluated for each cell system. In each case, the optimum charge ratio for a given reagent was used for the comparison with nucleofection. The nucleofection buffer and program are critical parameters for nucleofection, so different programs and buffers were tested to obtain the optimal transfection efficiency.

## Methods

### Cells and Culture Conditions

#### Adherent Cells

Primary embryonic pig fibroblasts (P16) (obtained from Dr José Cibelli, Michigan State University, East Lansing, MI) and embryonic rabbit ear fibroblasts (REF) (obtained from Dr Fuliang Du, University of Connecticut, Storrs, CT) [14] were grown in Dulbecco's Modified Eagle's Medium (DMEM) supplemented with 15% or 10%, respectively, fetal calf serum (FCS, Hyclone), 2-mercaptoethanol (1.5%), and glutamine (2 mM). Sickle cell disease (SCD) transgenic mouse embryonic stem cells (MESCs) containing a YAC carrying 240 kB of the β^S^-globin locus (obtained from Dr YW Kan, University of California, San Francisco, CA) were grown on gelatin coated plates on a mitomycin C inactivated SNL mouse embryo fibroblast feeder layer expressing leukemia inhibitory factor (LIF) in DMEM containing and 15% FCS (Hyclone), 2 mM glutamine (Invitrogen), 10^-4 ^M non-essential amino acids (Invitrogen), 10^4 ^M 2-mecaptoethanol (Sigma-Aldrich) [15]. Immortalized human bronchial epithelial cells (16HBE14o- [16,17] and CFBE41o- [18-20]) cells and the adenovirus 5 immortalized human embryo kidney cell line, HEK 293 [21], (American Type Tissue Culture Collection, Manassas, VA) were grown on tissue culture plastic coated with an extra-cellular matrix cocktail comprised of human fibronectin (FN) (BD laboratories, NJ), Vitrogen (V) (BD laboratories), and bovine serum albumin (BSA) (Biosource/Biofluids, Camarillo, CA) (FN/V/BSA) in Minimum Essential Medium (MEM) supplemented with 10% FCS, 1% (v/v) glutamine, 1% pen/strep [22]. Primary pig and human tracheal epithelial (PTE and HTE, respectively) cells (obtained from Dr J H Widdicombe, University of California, Davis, CA and Dr W E Finkbeiner, University of California, San Francisco, CA) were grown in modified LHC8e medium (MLHC8e): LHC8 medium (Biosource/Biofluids) supplemented with 2 mM glutamine, 1 ml Stock 4 solution (Biosource/Biofluids), 2 μg/ml insulin (Biosource/Biofluids), 1 ml Trace Elements solution (Biosource/Biofluids), and epinephrine (0.5 μg/ml) (Biosource/Biofluids) [22]. All cells were grown at 37°C in humidified air containing 5% CO_2 _and subcultured every 2-3 days by trypsinization.

#### Non-adherent Cells

SC1 lymphoblasts (American Type Tissue Culture Collection, Manassas, VA, ATCC#CRL-8756) were homozygous for the sickle cell allele) and LT1-1B1 human lymphoblasts with a G>C substitution mutation in exon 3 in *HPRT1 *gene (codon 51) [23]. SC1 cells were grown in suspension culture in RPMI 1640 medium supplemented with 20% Fetal Calf Serum (ATCC) with routine media changes every 48 h. LT1-1B1 cells were also grown in RPMI 1640 medium but supplemented with 10% FBS (Sigma, St Louis, MO), 5 mM L-glutamine, 40 mM HEPES, and 10 mM 6-thioguanine (6TG) (Sigma, company info). Hematopoietic CD34^+ ^cells were isolated from human fetal liver (obtained from Dr M Meunch, University of California, San Francisco, CA) and grown as described previously [24] in serum-free culture medium consisting of Iscove's modified Dulbecco's medium (IMDM) (Sigma Chemical, St. Louis, MO) supplemented with 7.5 10-5 α-thioglycerol (Sigma Chemical), 50 μg/ml gentamicin, 2% fraction-V ethanol-extracted BSA (Boehringer Mannheim Biochemicals, Indianapolis, Indiana, USA), 200 μg/ml human iron-saturated transferrin (Boehringer Mannheim Biochemicals), 10 μg/ml recombinant human insulin (Boehringer Mannheim Biochemicals), and 20 μg protein/ml human low density lipoprotein (Sigma Chemical), 10 U/ml erythropoietin (Amgen, Thousand Oaks, CA), and 50 ng/ml c-kit ligand (KL) (R&D Systems Inc., Minneapolis, MN). Cells were grown under humidified conditions in 5% CO_2 _with media changes every 48 h.

All cells were obtained with the appropriate IRB and IACUC approvals at the institutions where they were generated. The human samples were obtained in accordance with the Helsinki Declaration http://www.wma.net/en/30publications/10policies/b3/index.html from autopsy material with informed consent when samples had identifiable markers. When samples were anonymous, informed consent was not required for autopsy materials or discarded tissue. Human fetal livers were obtained from mid-gestation fetuses after maternal consent from elective abortions. Research with fetal tissue and human tracheal epithelial cells obtained from autopsy were performed with approval of the Committee of Human Research at the University of California, San Francisco under approvals H8858-18760-04/05 and H493-27303-04, respectively.

### Nucleofection

In the electroporation (nucleofection) experiments, 1 - 2 × 10^6 ^cells were resuspended in 100 μl of transfection buffer (Table 1). The pmaxGFP plasmid (AMAXA Biosystems, Gaithersburg, MD) that contains an enhanced green fluorescent protein (*EGFP*) gene under regulation of a cytomegalovirus (CMV) enhancer/promoter element and is kanamycin resistant, was then added (2 μg/transfection sample) to the cell suspension. The cell/DNA mixtures, in 1 cm transfection cuvettes, were nucleoporated according to a specific predefined program. Following the electroporation, the cells were incubated in their respective culture medium pre-heated to 37°C for 10 min, and then seeded into cell type-specific growth medium. Unless otherwise indicated all nucleofection experiments were carried out in triplicate using 3 separate dishes for each point.

**Table 1:** Cells and Optimal Nucleofection Conditions

Species	Cell name	Cell description	AMAXA program	AMAXA buffer
Pig	P16	Pig Fetal Fibroblasts	U-20	NHDF
	
	PTE	Primary Pig Tracheal Epithelial Cells	T-20	Basic epithelial cell

Human	16HBE41o-	Immortalized Human Bronchial Epithelial cell Line (WT)	O-17	V
	
	CFBE41o-	Immortalized Human CF Bronchial Epithelial Cell Line (ΔF508/ΔF508))	O-17	V
	
	HTE	Primary Human Tracheal Epithelial Cells	T-20	Basic epithelial cell
	
	LT1-1B1	Immortalized Human Lymphoblasts (HPRT mutant)	G-16	T
	
	SC-1	Immortalized Human Lymphoblasts (β^S^-globin mutant)	G-16	T
	
	HSPC	Primary Hematopoietic Stem/Progenitor Cells (CD34+ lin-)	U-08	CD34+
	
	HEK 293	Adenovirus immortalized human embryonic kidney cells	X-01	V

Rabbit	REF	Rabbit Ear Fibroblasts	U-23	NHDF

Mouse	MESC	Transgenic mouse embryonic stem cells (β^S^-globin mutant)	A-24	Mouse ES cell

The table contains the cells used in the studies and their origin. For each cell line/type, the optimal nucleofection program and buffer are indicated.

The MESCs were separated from the SNL feeder cells by short-term (30 min) plating of the trypsinized mixed cell population in Petri dishes not coated with gelatin. The SNL fibroblasts preferentially adhere and the MESCs are readily harvested for nucleofection.

### Transfection with Chemical Reagents

Before transfection 3 - 5 × 10^5 ^cells were seeded into individual wells of 6 well plates. After a 24 h incubation in growth medium, the cells were exposed to the polyplexes or lipoplexes that each contained 2 μg pmaxGFP plasmid/well of cells. Each transfection was carried out in triplicate and repeated 2 to 3 times. Following transfection the cells were incubated at 37°C in humidified-air (5% CO_2_) for 2 h. The transfection medium was then removed and the cells were incubated for an additional 48 h in complete medium (2 ml per well).

#### Lipofectamine 2000 and Lipofectamine Plus

Plasmid DNA and Lipofectamine 2000 (Invitrogen, Carlsbad, CA) were diluted in two independent 250 μl volumes of Opti-MEM reduced serum medium (Invitrogen) without serum and mixed gently. For Lipofectamine Plus transfections, the DNA was pre-incubated with 4 μl of Plus reagent and Opti-MEM to a final volume of 25 μl. After a 5 min incubation at room temperature, the DNA and the Lipofectamine 2000 in Opti-MEM were combined and incubated for an additional 20 min at room temperature to allow the DNA-Lipofectamine 2000 complexes to form. The DNA- Plus mix (25 μl) was added to an equal volume the Lipofectamine 2000 reagent mixed with Opti-MEM and incubated for an additional 30 min at room temperature. The DNA-Lipofectamine 2000 complexes were then added to each well containing cells and medium. The vol/wt ratios of Lipofectamine 2000/DNA were: 3/1, 5/1 and 7/1, and 1/1 for Lipofectamine Plus/DNA.

#### Polyethylenimine (PEI)

PEI (QBiogene, Morgan Irvine, CA) and plasmid DNA were each diluted with equal volumes of 150 mM NaCl. The DNA solution was then added to the PEI solution, and after a 20 min incubation at room temperature, 200 μl/well aliquots of the DNA-PEI complexes were added to cells grown in serum containing medium in individual wells. The charge ratios (+/-) of PEI nitrogen residues/DNA phosphates were: 3/1, 5/1 and 8/1

#### Effectene

Effectene transfections were conducted according to the manufacturer's instructions (Qiagen, Valencia, CA). The vol/wt ratios of Effectene/DNA were 10/1 and 25/1.

### Analysis of transfected cells

Cells were harvested 48 h post-transfection, washed, and resuspended in PBS. In adherent cell cultures, only cells adhering to the culture dish before trypsinization were counted as viable. Cells in suspension were exposed to PBS containing 0.02% EGTA and 1 μg/ml propidium iodide to identify the nonviable cells through propidium iodide fluorescence. The cells were then sorted by flow cytometry, evaluated with the Cellquest software (BD Biosciences, San Jose, CA) to determine the proportion of fluorescent cells.

The cells were transfected with a reporter plasmid encoding the EGFP using either nucleofection or four different chemical reagents (Effectene, Lipofectamine 2000, Lipofectamine Plus and PEI). Transfection efficiency was determined 48 h after transfection as the:

The percent cytotoxicity following transfection was:

Where B = the # of adherent or total # of cells when grown in suspension, in the transfected sample at the time of harvest, C = # of nontransfected adherent or total # of cells when grown in suspension, present at the time of harvest, and T is toxicity.

Cell viability is therefore the number of viable transfected cells present at the 48 h post-transfection harvest time compared to control, non-transfected cells, i.e., the percent viability (V) is:

This proportion of live cells present at the time of harvest was taken to be an indicator of relative cell cytotoxicity and consequently, the cell viability following transfection.

## Results

### Nucleofection

#### Pig and Rabbit Fetal Fibroblasts

**Figure 1:**
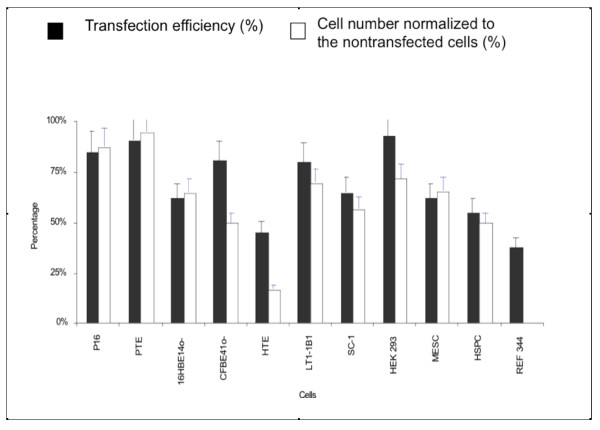
**The transfection efficiency obtained 48 hours after nucleofection of 10^6 ^cells with 2 μg of pmaxGFP plasmid**. The cells are described in Table 1. The error bars represent the standard error of the mean (SEM), with n = 3.

The ability to generate transgenic animals through somatic cell nuclear transfer (SCNT) has opened up many possibilities for the study of disease and the development of therapies [25]. Pig fetal fibroblasts (P16) previously used for SCNT (J Cibelli, personal communication) were transfected using 30 different nucleofection programs in combination with the AMAXA NHDF buffer to determine the optimal parameters for nucleofection. Program U-20 was the most effective and resulted in a 90% efficiency of GFP expression and 5% cytotoxicity (Figure 1). The most effective program/buffer combination for rabbit embryo fibroblasts (REF) transfection was program U-23 with the NHDF buffer (Table 1). After 48 h, GFP expression was observed in 38% of the cells (Figure 1).

#### Human and Pig Primary Tracheal Epithelial cells

Primary airway epithelial cells play a crucial role in the study of airway disease and infection. The ability to efficiently transfer of genes into these cells is critical in evaluating the mechanisms underlying airway epithelial cell function and airway disease pathology. Because there was no optimized protocol available for nucleofection of primary human or pig tracheal epithelial cells, 3 different buffers were tested (EP-39, EP-42 and E-58 (Basic Epithelial Cell buffer)). Optimization of the human tracheal epithelial (HTE) cells involved pairing each buffer with 9 different programs. The optimal transfection efficiency was achieved using program T-20 and the Amaxa Basic Epithelial Cell buffer and resulted in 47% expression efficiency and 83% cytotoxicity (17% viability) (Figure 1, Table 1).

Nucleofection of primary pig tracheal epithelial (PTE) cells under the same conditions, i.e., using the same buffer and program, gave a transfection efficiency of 90%. The 5% cytotoxicity (95% viability) of the transfected PTE cells detected 48 h after transfection was considerably less than that observed with the HTE cells (Figure 1)

#### Human Bronchial Epithelial Cell Lines

Immortalized bronchial epithelial cells [26-28] were studied, because they are routinely used as models of cystic fibrosis (CF) and airway disease. Normal, 16HBE14o- [16], and CF, CFBE41o- [17-20], cell lines were optimally transfected with buffer V and program O-17 (Table 1). The 16HBE14o- cells showed a 62% viability and 65% transfection efficiency, while transfection of the CFBE41o- cells gave 81% expression efficiency at 50% viability (Figure 1).

#### Hematopoietic Stem/Progenitor Cells

Hematopoietic stem/progenitor cells (HSPCs) are attractive targets for gene delivery and therapy because of their potential for self-renewal and multilineage differentiation [29,30]. These properties make them ideally suited for *ex vivo *gene transfer that could result in a treatment for numerous inherited and/or hematologic disorders.

HSPCs isolated from fetal liver [24] were nucleofected using Amaxa CD34 buffer and program U-08 (Table 1). GFP was expressed in 55% of the HSPCs accompanied by a viability of 50%. Furthermore, the ability of the HSPCs to differentiate into red blood cells persisted after transfection when the cells were grown in differentiating medium (R Maurisse and DC Gruenert, unpublished data).

#### Lymphoblasts

Epstein-Barr virus (EBV) transformed lymphocytes (lymphoblasts) were nucleofected with buffer T and program G-16 (Table 1). The transfection efficiency of two different lymphoblast lines (SC-1 and LT1-1B1) was 75% with an 80% viability (Figure 1).

#### Mouse Embryonic Stem Cells

Transgenic mouse embryonic stem cells (MESCs) that contain a YAC that carries 240-kb β^S^-globin gene family [15] were optimally transfected using the Amaxa MESC buffer with program A-24 (Table 1). The transfection efficiency and viability was 62% and 66%, respectively (Figure 1). The cells were not effectively transfected using chemical reagents due to high cytotoxicity and/or senescence following reagent exposure (H Emamekhoo and DC Gruenert, unpublished observations).

#### HEK 293 Cells

The HEK 293 (human embryonic kidney) cell lines was nucleofected with Amaxa buffer V and program X-01 (Table 1). The efficiency of transfection and the viability were 93% and 72%, respectively (Figure 1).

### Nucleofection vs Chemical Transfection

**Figure 2:**
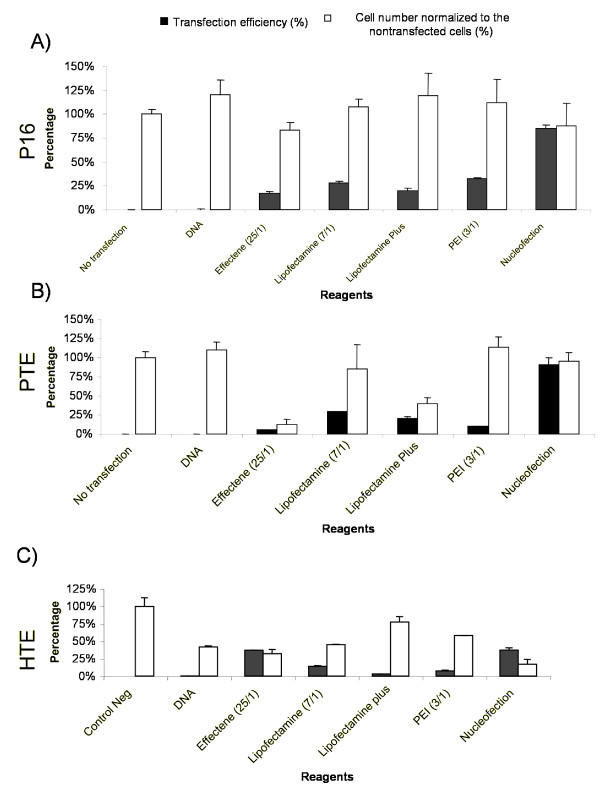
**Comparison of the transfection efficacy of pmaxGFP with chemical reagents (Effectene, Lipofectamine 2000, Lipofectamine Plus, and PEI) and nucleofection**. The vol/wt ratios (μl reagent/μg DNA) for Effectene and Lipofectamine 2000 transfection and the (+/-) charge ratios (PEI nitrogen residues/DNA phosphates) for PEI transfection are indicated in parentheses. Transfection efficacy is indicated by the black bar, and the relative number of adherent cells in the transfected cells was compared to the number in nontransfected control cultures is indicated by the white bar for (A) pig fetal fibroblast (P16), (B) primary pig tracheal epithelial (PTE) cells, and (C) primary human tracheal epithelial (HTE) cells. The error bars reflect the standard error of the mean (SEM), with n = 3.

A number of chemical reagents were used to transfect 5 × 10^5 ^cells with 2 μg of pmaxGFP plasmid. The transfection efficiencies and the viabilities were then compared to those observed for nucleofection of the same cell lines/types (Figure 2). The quantity of plasmid per cell transfected with the chemical transfection reagent was two-fold more than that used for nucleofection. For each reagents one to three reagent/DNA ratios were tested either as a ratio of vol/wt (μl reagents/μg DNA); Effectene: 10/1 and 25/1; Lipofectamine 2000: 3/1, 5/1 and 7/1; Lipofectamine Plus 1/1. The reagent/DNA ratios evaluated for PEI were based on positive and negative charges. The charge ratios (Nitrogen residues/Phosphate) evaluated was: 3/1, 5/1 and 8/1 PEI.

Only the optimal, i.e., in terms of transfection efficiency, reagent/DNA ratios were compared (Figure 2). The data presented compares the relative effectiveness of plasmid delivery into pig fetal fibroblast (P16) as well as primary human and pig tracheal epithelial cells (HTE and PTE, respectively) by chemical reagents and nucleofection.

#### Pig Fetal Fibroblasts

P16 cells were transfected with 2 μg of pmaxGFP plasmid. The transfection efficiencies were 18% (Effectene 25/1), 28% (Lipofectamine 2000; 7/1), 20% (Lipofectamine Plus) and 32% (PEI; 3/1) (Figure 2-A). Transfection by nucleofection gave an efficiency of 85%.

#### Pig Tracheal Epithelial Cells

PTE were transfected with 2 μg pmaxGFP plasmid in a complex with Effectene, Lipofectamine 2000, Lipofectamine Plus and PEI (Figure 2-B). The transfection efficiencies of the PTE cells were 5% (Effectene; 25/1), 30% (Lipofectamine 2000; 7/1), 21% (Lipofectamine Plus) and 10% (PEI; 3/1) transfection respectively at the ratio indicated (Figure 2). The nucleofection resulted in a transfection efficiency of 90% and cytotoxicity of 5%.

#### Human Tracheal Epithelial Cells

HTE cells were transfected with the four reagents indicated below and by nucleofection (Figure 2-C). The transfection efficiencies obtained were: 37% (Effectene; 25/1), 14% (Lipofectamine 2000; 7/1), 3% (Lipofectamine Plus; 1/1), and 8% (PEI; 3/1), respectively (Figure 2C). Nucleofection gave a transfection efficiency of 45%.

#### HEK 293 Cells

The transfection efficiency and viability with Lipofectamine 2000 was 98% and 67%, respectively. The transfection efficiency with Lipofectamine Plus was 82% with a viability of 80% (data not shown).

## Discussion

The delivery of genes into primary and immortalized cell lines is an underpinning of mammalian molecular biology and has become increasingly important in biomedical research and therapeutic development. Defining the parameters necessary for transfection optimization is, thus a critical element in further enhancing gene delivery efficacy in a wide range of cells. While there has been significant work done in the development of chemical and viral reagents for the delivery of recombinant DNA, only limited improvements have been made in physical delivery systems [1]. The development of a novel electroporation system by AMAXA has shown considerable promise as a system for delivering DNA to a broad range of cell lines and cell systems that grow either as adherent monolayers or in suspension [7,31-34]. A number of cell lines from human and animals that have been particularly important for characterization of airway diseases such as cystic fibrosis and asthma, for somatic cell nuclear transfer, for the study of hematopoietic diseases, and for mutation analysis were evaluated and compared for their ability to be efficaciously transfected with the nucleofection system. With the exception of HEK293 cells, when compared to chemical DNA delivery vehicles, nucleofection appears to be, in general, more effective and less toxic. The transfection efficiency and toxicity is equivalent following nucleofection or Lipofectamine transfection of HEK293 cells (Figure 1).

Transfection of two immortalized human airway epithelial cell lines, 16HBE14o- and CFB41o- and primary airway epithelial cells from pig and human (PTE and HTE, respectively) showed that nucleofection was more effective than the four chemical reagents tested with the exception of the HTE cells that were also effectively transfectable with Effectene. Primary human airway epithelial cells were difficult to transfect even by nucleofection (45%) when compared to the PTE (95%). While the reason for this difference is not certain, it is possible that cells at different passages or in different stages of differentiation will have varying responses to insult. Additional studies will need to be undertaken to determine whether the transfection efficiency and viability following nucleofection can be further enhanced.

The development of somatic cell nuclear transfer using fetal fibroblast as donor cells has played a central role in the cloning of animals such as the pig and the rabbit [14,35-37]. Greater than 95% of the P16 cells expressed GFP following nucleofection while the rabbit ear fibroblasts (REF) appeared to be more recalcitrant to transfection and gave a GFP expression frequency in the range of 40%. This difference may be due to species-specific factors that affect the transport and/or of expression the plasmid DNA in the cell nucleus. In addition, differences in the age of the cultured cells, and cell density may also play a factor. These elements need to be considered when optimizing transfection conditions and should be addressed empirically.

Suspension cultures of hematopoietic origin have been notoriously difficult to transfect with chemical reagents and have had to rely on viral vector systems to facilitate DNA delivery [1]. However, the studies here showed that nucleofection was able to transfect both primary human hematopoietic stem/progenitor cells as well as immortalized lymphoblasts giving levels GFP expression in the range of 60-80% with relatively low levels of cytotoxicity. Thus, nucleofection may be an effective means of *ex vivo *genetic modification of hematopoietic stem cells that have multilineage potential.

Embryonic stem (ES) cells have become increasingly more important due their potential for organ regeneration and for the development of models to study disease. Mouse ES cells (MESCs) have been notoriously difficult to transfect with chemical reagents, and have thus been relegated to transfection by electroporation. Standard electroporation protocols have resulted in high levels of cytotoxicity that have undermined the ability to transfer genes into the cells and the potential of the MESCs to produce viable embryos or differentiate in a lineage directed fashion. The nucleofection system has provided the opportunity to overcome some of these issues by enhancing transfection efficacy and MESC viability. As indicated by the studies presented here, MESCs can be routinely transfected at efficiencies of about 60% with a concurrent 60% viability. These observations have important implications for the transfection of human ES cells and for their genetic modification and directed differentiation in that nucleofection has the potential of producing genetically modified cells that can be phenotypically manipulated without losing their pluripotency.

## Conclusion

This study demonstrates the nucleofection system is effective for a broad range of cell lines and cell types, resulting in high levels of transgene expression and low toxicity. Not only is it superior when compared to various commercially available chemical DNA delivery vehicles in terms of transfection efficacy and viability, it also has potential therapeutic applications in *ex vivo *gene delivery.

## Abbreviations

ATCC: American Type Tissue Culture Collection; CF: cystic fibrosis; CFBE: CF bronchial epithelial; CMV: cytomegalovirus; DMEM: Dulbecco's modified Eagle's medium; EBV: Epstein-Barr virus; EGFP: enhanced green fluorescent protein; ES: embryonic stem; FCS: fetal calf serum; FN/V/BSA: fibronectin/Vitrogen/bovine serum albumin; HSPC: hematopoietic stem/progenitor cells; HBE: human bronchial epithelial; HEK: human embryo kidney; HPRT: hypoxanthine phosphoribosyl transferase; HTE: human tracheal epithelial; IMDM: Iscove's modified Dulbecco's medium; KL: c-kit ligand; LIF: leukemia inhibitory factor; MEM: minimal essential medium; MESC: mouse ES cells; PBS: phosphate buffered saline; PEI: polyethylenimine; PTE: pig tracheal epithelial; REF: rabbit embryo fibroblasts; SCD: sickle cell disease; 6-TG: 6 thioguanine; YAC: yeast artificial chromosome.

## Authors' contributions

RM - designed and conducted experiments with epithelial cells, HSPCs and calibrated Amaxa system and EGFP analysis, analyzed and compiled data, wrote initial draft of manuscript. DD - designed and conducted experiments with REF and transformed cells, analyzed data, edited manuscript. HE - designed and calibrated experiments with HSPCs and assisted with HEK and lymphoblast studies, analyzed data. BB - designed and calibrated experiments with LT1-1B1 lymphoblasts, analyzed data. AA - designed and calibrated experiments with SC-1 lymphoblasts, analyzed data. HP - designed and conducted experiments with HEK cells, analyzed and compiled data. DCG - designed entire project, coordinated research efforts, analyzed data, wrote and edited manuscript, finalized manuscript. All authors have read and approved of the final manuscript.
